# Early exposure to antibiotic drugs and risk for psychiatric disorders: a population-based study

**DOI:** 10.1038/s41398-019-0653-9

**Published:** 2019-11-26

**Authors:** Catharina Lavebratt, Liu L. Yang, MaiBritt Giacobini, Yvonne Forsell, Martin Schalling, Timo Partonen, Mika Gissler

**Affiliations:** 1 Karolinska Institutet, Department of Molecular Medicine and Surgery (MMK), Stockholm, Sweden; 2 Karolinska University Hospital Solna, Center for Molecular Medicine, Stockholm, Sweden; 3 PRIMA Child and Adult Psychiatry, Stockholm, Sweden; 4 Karolinska Institutet, Department of Public Health Sciences, Stockholm, Sweden; 5 National Institute for Health and Welfare (THL), Department of Public Health Solutions, Helsinki, Finland; 6 Department of Neurobiology, Care Sciences and Society, Karolinska Institutet, Stockholm, Sweden; 7 University of Turku, Research Centre for Child Psychiatry, Turku, Finland

**Keywords:** ADHD, Depression

## Abstract

Early life exposure to infection, anti-infectives and altered immune activity have been associated with elevated risk of some psychiatric disorders. However, the risk from exposure in fetal life has been proposed to be confounded by familial factors. The hypothesis of this study is that antibiotic drug exposure during the fetal period and the first two postnatal years is associated with risk for later development of psychiatric disorders in children. All births in Finland between 1996 and 2012, 1 million births, were studied for antibiotic drug exposure: mothers during pregnancy and the children the first two postnatal years. The children were followed up for a wide spectrum of psychiatric diagnoses and psychotropic drug treatment until 2014. Cox proportional hazards modeling was used to estimate effects of antibiotic drug exposure on offspring psychiatric disorders. Modestly (10–50%) increased risks were found on later childhood development of sleep disorders, ADHD, conduct disorder, mood and anxiety disorders, and other behavioral and emotional disorders with childhood onset (ICD-10 F98), supported by increased risks also for childhood psychotropic medication. The prenatal exposure effects detected were not explained by explored familial confounding, nor by registered maternal infections. To conclude, this longitudinal nation-wide study shows that early life antibiotic drug exposure is associated with an increased risk for childhood development of psychopathology. Given the high occurrence of early-life antibiotic exposure, these findings are of public health relevance. Whether the associations reflect effects of the antibiotic drug use or of the targeted infections remains to be explored further.

## Introduction

Certain parasite, viral and severe bacterial infections mainly in hospitalized cases have been shown to increase the risk for psychotic disorders and mood disorders^1–3^. A few cross-sectional studies in adults have reported that also certain non-hospitalized infection events were associated with an overrepresentation of psychotic, mood or anxiety disorders^4–6^. Further, nation-wide population-based registry studies in Denmark reported elevated risks for a spectrum of mental disorders after infections and anti-infective drug exposures, especially antibiotics, in childhood/adolescence^7,8^. There are reports of an increased peripheral immune activation in most psychiatric disorders in pediatric populations^9^, and the peripheral immune system is being recognized for its importance in neurodevelopment^10^.

Very early life, ie the prenatal period and infancy, may imply a more pronounced vulnerability to the exposure due to the ongoing complex neurodevelopmental events including, but not limited to, neurogenesis, axonal and dendritic growth, synaptogenesis, and refinement of these synaptic connections^11^. Maternal infections and inflammation during pregnancy have been reported to affect the fetal brain, increasing the risk of offspring mental disorder with a focus mainly on psychotic disorders^3,12–14^. However, not all fetal exposure studies considered potential confounders e.g. maternal psychiatric diagnoses or socioeconomic factors. In a recent nation-wide registry study of a Danish birth cohort, maternal use of anti-infective agent during pregnancy was after adjustment for maternal and birth-related factors associated with an increased risk of any offspring psychiatric disorder, with a higher risk estimate than that of paternal anti-infective agent use. However, the risk estimates were similar for exposure during and outside the pregnancy period suggesting that the association was explained by unmeasured confounding^15^. On the other hand, a large cohort study in New Zeeland reported association for first year’s antibiotic exposure with behavioral difficulties and mood symptoms^16^, while no association between exposure during the first two postnatal years and autism and attention-deficit hyperactivity disorder (ADHD) was reported from registry studies in Canada^17,18^.

A putative association between antibiotic drug exposure and later psychiatric disorder might not only reflect the targeted bacterial infection and immune response thereto. Antibiotic treatment generally disrupts the bacterial microbiota in the intestine and other barriers, which in early developmental windows has resulted in marked behavioral effects in rodents, e.g. depressive-like and anxiety-like behaviors and cognitive changes^19–22^. The induced changes in barrier microbiota open up for opportunistic infections. Specifically the gut-brain axis works through modulation of brain activity via neural pathways and endocrine and immune mechanisms^23–25^. Thus, it is possible that the instability and immaturity of the bacterial microbiota during the first years of life imply a vulnerability to antibiotic drug exposure that could increase the risk for altered barrier microbiota, opportunistic infections and psychiatric disorder later in life^26–29^.

The aim of this study was to investigate if exposure to antibiotics, prenatally and in the first 2 years of life, influences the risk for a wide spectrum of psychiatric disorder up to 18 years of age using Finnish nation-wide registers.

## Methods

### Study population and data sources

All pregnancies ending in live birth in Finland between 1996 and 2012 were identified using the Drugs and Pregnancy database^30^ and included 990,098 births (Supplementary Data, Tables S1 and S2). There were no exclusion criteria. The Drugs and Pregnancy database is derived from the Medical Birth Register (MBR), the Register on Induced Abortions and the Register of Congenital Malformations, all currently kept at the Finnish National Institute for Health and Welfare (THL). MBR includes information since 1987 on all live births and stillbirths in Finland with a gestational age (pregnancy length) of 22 weeks or more, or with a birth weight of 500 g or more. The MBR data are supplemented from birth and death certificates and are complemented from the Cause-of-Death Register and from the maternity hospital records. According to data quality studies of the MBR, the majority of the register content corresponded well or satisfactorily with hospital record data^31^. Information on maternal and offspring (child) drug purchases was obtained from the Finnish Register on Reimbursement Drugs (RRD) maintained by the National Social Insurance Institution (SII). The Nordic countries are in a unique position concerning statistics on the consumption of medicines with registries showing high validity^32^. In Finland, all antibiotic drugs are prescription-only medicines. Prescription-only medicines are sold only in pharmacies, and dispensing requires a prescription issued by a physician or a dentist. All Finnish citizens are covered with health insurance and the use of health care services are free of charge or heavily subsidized. All Finnish citizens and permanent residents are entitled to reimbursement of prescribed medicine^33^. The RRD automatically registers all reimbursed drug prescriptions (Anatomical Therapeutic Chemical (ATC) codes) that were since 1996 dispensed at pharmacies. The Drugs and Pregnancy database includes all RRD records of purchases during pregnancy. Thus, the information on maternal drug purchases during pregnancy, stratified in trimesters T1, T2, and T3, were obtained from the Drugs and Pregnancy database, while the offspring purchases were obtained directly from RRD.

Maternal and offspring medical diagnoses, classified according to the WHO International Classification of Diseases (ICD), were obtained from the Finnish Care Registers for Health Care (HILMO), which covers adult psychiatric diagnoses well according to validation studies^34^ and one study reported good validity for pediatric ASD in HILMO^35^. Information from the registers was linked through the unique personal identification numbers assigned to all Finnish citizens and permanent residents, as set out in the permission from the register administrators (SII and THL). The Drugs and Pregnancy-database steering committee and the data protection authority in Finland authorized this study. The registered women and their children were not contacted, and informed consents were therefore not required.

### Definition of exposures: in utero (fetal) and offspring postnatal antibiotic treatment

The exposure was based on purchases of antibiotic drugs in ten ATC codes J01A-J01X (A, B, C, D, E, F, G, M, R, X). The analysis included (a) any antibiotic drug (J01A-X), and (b) antibiotic drugs categorized into (i) “airway antibiotics”, i.e. those against gram-positive bacteria commonly used to treat respiratory infections (J01C, D and F) and (ii) “urinary tract antibiotics”, i.e., those against urinary tract or skin or soft tissue infections (J01E and M). A separate analysis included broad-spectrum (J01A, B, E, F, G, M, R, X) and narrow spectrum (J01C) antibiotics. For fetal exposure, mothers’ purchases during pregnancy were used. Offspring exposure to antibiotics postnatally was stratified: the first 6 months, 6–11 months, 1–2 years.

Information on offspring in-patient care for infectious disorder, previously shown to commonly be treated with antibiotics^36^, was retrieved from HILMO: ICD10 A00-B99, G00-G03, J00-J22, K35, L08, N10, and N30 as primary or secondary diagnoses. Maternal in- and out-patient data on infections during pregnancy were obtained from HILMO and MBR: ICD10 A00-B99, J00-J22, K35, L00-08, and O23.

### Definition of outcomes: offspring psychiatric disorders

Data on psychiatric disorders for the offspring, as primary or secondary diagnoses between birth and December 2014, was obtained from HILMO. The diagnosis-groups indicated by the following ICD–10 codes were studied: F20–39, F92 (Mood disorders), F40–43, F93 (Anxiety disorders), F50 (Eating disorders), F51 (Non-organic sleeping disorders), F84 (autism), F90–F91 (ADHD and Conduct disorders) and F98 (Other behavioral and emotional disorders with onset usually occurring in childhood and adolescence) (Supplementary Table S1).

Information on prescription of psychotropic drugs to offspring was obtained from the RRD and used as outcome in a second model: ATC code N05 (antipsychotics, anxiolytics, hypnotics and sedatives), N06A (antidepressants) and N06B (psychostimulants and nootropics).

### Other variables used as covariates

Information on offspring birth year, sex, perinatal problems (birth before gestational week 37, birth weight < 2500 grams, or small birth weight or length for the gestational age according to Finnish sex-specific standards^37,38^), number of fetuses, mode of delivery, maternal age at delivery, parity, the mother’s country of birth and marital status, and maternal smoking, were obtained from the Drugs and Pregnancy database. Smoking and marital status correlate highly with socioeconomic status^39^. Data on the mother’s diagnoses related to systemic inflammatory disorders (ICD–10: M30–M36 in 1996–2014, i.e., systemic connective tissue disorders) and previous in-patient care due to mental health disorders (ICD–8: 290–317 in 1969–1986, ICD–9: 290–319 in 1987–1995, and ICD–10: F00–F99 in 1996–2014), as primary or secondary diagnoses, was obtained from HILMO.

### Statistical analysis

Cox proportional hazards modeling was used to estimate the effect of antibiotic drug exposure, in utero and postnatally, on the outcomes (i) offspring psychiatric diagnosis and (ii) offspring prescription of psychotropic drug. As the different neurodevelopmental events have certain time-windows^11^, and the gut microbiota develops extensively during the first 2 postnatal years^25^, the analyses measuring postnatal antibiotic drug exposure were stratified for age groups: first 6 months, 6–11 months, 1–2 years. The same covariates were adjusted for (see Table 1) in both the fetal and the postnatal exposure models because of possible confounding^26,40–43^, except for that the postnatal exposure model was adjusted also for mother’s purchases of antibiotics during pregnancy [yes/no].

**Table 1:** Adjusted Cox hazard ratios (HR) and 99% confidence intervals (CI) for the outcomes (columns) (i) psychiatric disorder (ICD-10 F diagnoses), and (ii) psychotropic medication until 2014, in relation to exposure to antibiotics (rows) in fetal life, trimesters 1–3 (i.e. maternal purchase of antibiotic drug at least during pregnancy), among the 990 098 births (1996–2012). Outcomes after birth are considered.

Antibiotic drugs	Any F diagnosis		F30–39, F92		F40–43, F93		F50		F51		F84		F90–91		F98		Any psychotropic medication		N05		N06A		N06B	
ATC code groups	*n* = 105641		*n* = 19149		*n* = 27164		*n* = 3320		*n* = 5092		*n* = 7495		*n* = 19706		*n* = 21857		*n* = 56340		*n* = 37491		*n* = 13050		*n* = 16722	
J01CDF, J01EM, J01C or ABEFGMRX	HR	99% CI	HR	99% CI	HR	99% CI	HR	99% CI	HR	99% CI	HR	99% CI	HR	99% CI	HR	99% CI	HR	99% CI	HR	99% CI	HR	99% CI	HR	99% CI
No, *n* = 735730	1.00		1.00		1.00		1.00		1.00		1.00		1.00		1.00		1.00		1.00		1.00		1.00	
Airway (CDF) *n* = 245664	1.16	1.14–1.18	1.17	1.12–1.22	1.19	1.15–1.23	1.13	1.02–1.26	1.40	1.29–1.51	1.05	0.98–1.13	1.21	1.17–1.27	1.21	1.17–1.26	1.17	1.14–1.20	1.17	1.13–1.20	1.15	1.10–1.21	1.20	1.15–1.26
Urinary tract, soft tissue (EM) *n* = 2070	1.20	1.02–1.40	1.41	1.02–1.97	1.30	0.97–1.74	0.94	0.36–2.49	1.15	0.55–2.42	1.49	0.88–2.52	1.53	1.11–2.11	1.15	0.81–1.65	1.18	0.95–1.46	1.13	0.86–1.48	1.09	0.69–1.72	1.29	0.88–1.88
CDF and EM^a^ *n* = 2470	1.18	1.02–1.36	1.15	0.82–1.61	1.43	1.11–1.85	1.08	0.46–2.54	1.51	0.84–2.74	0.99	0.55–1.78	1.00	0.70–1.44	1.22	0.89–1.68	1.08	0.88–1.32	1.11	0.87–1.43	1.01	0.66–1.55	0.90	0.60–1.35
Narrow spectrum (C) *n* = 240767	1.12	1.10–1.15	1.12	1.06-1.19	1.13	1.08–1.19	1.12	0.98–1.29	1.32	1.19–1.47	1.00	0.91–1.10	1.17	1.11–1.24	1.14	1.09–1.21	1.16	1.13–1.20	1.17	1.12–1.22	1.12	1.04–1.20	1.18	1.11–1.26
Broad spectrum (ABEFGMRX) *n* = 20651	1.19	1.14-1.24	1.36	1.24–1.48	1.26	1.17–1.36	1.13	0.90–1.43	1.41	1.17-1.70	1.05	0.89–1.25	1.29	1.17–1.41	1.29	1.17–1.41	1.14	1.07–1.20	1.14	1.06–1.22	1.28	1.15–1.42	1.22	1.10–1.35

Any F diagnosis was found for 10.7% of the cohort and included all ICD-10 F diagnoses

ICD-10 F30-39, F92 (1.9% of the cohort) Mood disorders; F40-43, F93 (2.7%) Anxiety disorders; F50 (0.34%) Eating disorders; F51 (0.51%) Nonorganic sleep disorders; F84 (0.76%) Autism Spectrum Disorder (ASD); F90-91 (2.0%) Attention Deficit Hyperactivity Disorder (ADHD) and Conduct disorders; F98 (2.2%) Other behavioral and emotional disorders with onset usually occurring in childhood and adolescence

Psychotropic medications studied defined according to the Anatomic Therapeutic Chemical (ATC) classification system were found for 5.7% of the cohort and included the following: ATC groups N05 (3.8%) antipsychotics, anxiolytics, hypnotics and sedatives; ATC group N06A (1.3%) antidepressants; ATC group N06B (1.7%) stimulants

Antibiotic drugs (ATC codes):

CDF: Any of J01C, D and/or F, i.e., airway antibiotics against primarily gram-positive bacteria, and no J01EM. EM: Any of J01E and/or M, i.e., urinary tract and soft tissue antibiotics against both gram-positive and gram-negative bacteria, and no J01CDF. J01C: Phenoxymethylpenicillin and penicillins with extended spectrum; J01D: Cephalosporins; J01F: Macrolides; J01E: Sulfonamides and trimethoprim; J01M: Fluoroquinolones.

Narrow spectrum: Only J01C

Broad spectrum: Any of J01A, B, E, F, G, M, R and/or X

Birth-related factors adjusted for: maternal age, parity, maternal smoking during pregnancy [yes/no], mother unmarried [yes/no], mother born elsewhere than Finland [yes/no], cesarean section [yes/no], mother’s inpatient care due to mental health disorders [yes/no], mother’s purchase of psychotropic drugs (N05 or N06) during pregnancy [yes/no], mother’s diagnoses related to systemic inflammatory disorders [yes/no], multiple birth [yes/no], offspring sex, perinatal health problems (birth weight < 2500 grams, gestational age < 37 weeks or small for gestational age according to Finnish sex-specific standards) [yes/no]

^a^Exposure to at least two antibiotic drugs

Five sensitivity analyses were performed: (1) Sibling pair analysis controlling for unmeasured familial confounding estimated the risk for the second sibling to have any F diagnosis or any psychotropic drug treatment after fetal exposure to any antibiotic drug (any J01). Mothers where only the first pregnancy was exposed were compared to mothers where both pregnancies were exposed. These Cox proportional hazards modeling sibling pair analyses were made for mothers exposed to any J01 during at least one of the two pregnancies, with mothers not exposed during any pregnancy as reference, adjusted for the above list of covariates as well as intra-pregnancy interval, and any F diagnosis or psychotropic medication in the first child. The first two subsequent singleton pregnancies of the same mother during the study period were included. The first child was not necessarily the first child of the mother. The siblings from both pregnancies were follow-up for psychiatric diagnoses or psychotropic medication until 2014. (2) For additional exploration of unmeasured confounding, offspring risks after any maternal J01 purchase was compared between the different exposure periods: only after pregnancy, only before pregnancy, and only during pregnancy. (3) Further, we estimated offspring risk after any maternal J01 purchase after adjustment for maternal infection during pregnancy. (4) In the postnatal exposure analysis, the offspring with an in-patient care for an infectious disorder, (ICD-10 A00-B99, G00-G03, J00-J22, K35, L08, N10, N30), were excluded, as these disorders were previously shown to commonly be treated with antibiotic drugs^36^, and the RRD does not cover in-patient care antibiotic treatment data. (5) These same in-patient care offspring with infections were included in the exposed group.

Hazard risk ratios (HRs) with 99% confidence intervals (CIs) were reported as measures of effect size. All statistical analyses were performed using SAS version 9.3 (SAS Institute Cary, USA).

## Results

### Description of the study population

All births in Finland between 1996 and 2012 were studied, being 990,098 births providing a longitudinal dataset with 9.7 million person-years. Characteristics of the birth cohort and pregnancy-related characteristics are described in Table S2. Of the births, 25.7% had reported use of any antibiotic drug any time during pregnancy (trimester T1 to T3). Maternal factors that associated with higher antibiotic use during pregnancy were smoking, previous inpatient care for psychiatric disorder or systemic inflammatory diagnoses and Finnish background (*p* < 0.001). From birth to 2 years of age, 81.7% of the offspring had at least one prescription of antibiotic drugs. The above maternal factors, as well as offspring male sex, was associated with higher offspring antibiotic use between birth and 2 years of age (*p* < 0.001). These factors were adjusted for in the following analyses. Of the offspring, 14.9% had no antibiotic exposure, neither prenatally nor during the first 2 years after birth, and 10.7% received a diagnosis of psychiatric disorder (ICD-10 codes F30–F51, and F84–F98, Supplementary Table S1) up to year 2014, that is the oldest offspring being 18 years (Table 1).

### Fetal exposure to antibiotic drugs

**Fig. 1:**
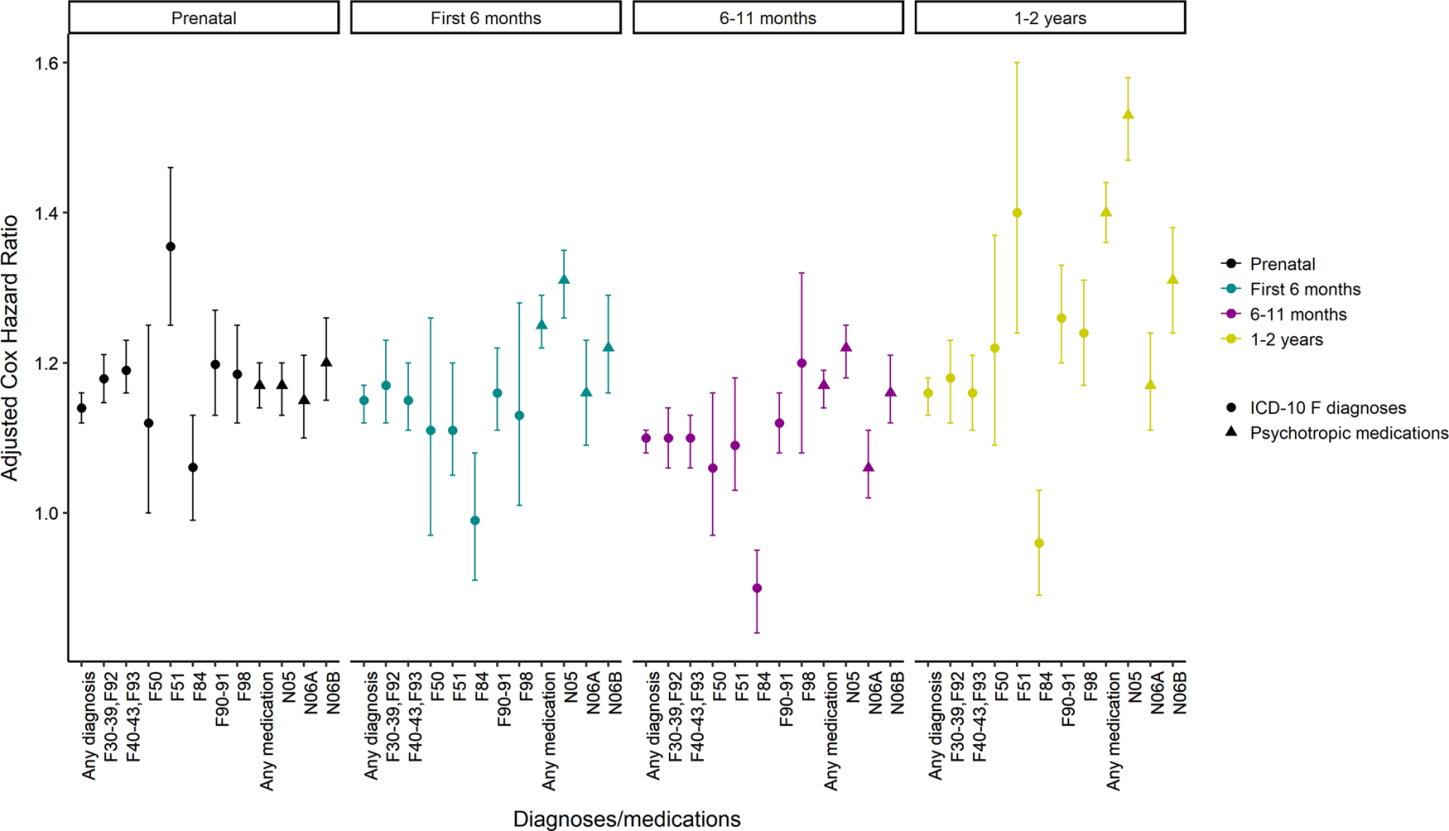
Overview of adjusted Cox Hazard Ratios for (i) psychiatric diagnoses (ICD-10 F diagnoses), and (ii) psychotropic medication, in relation to exposure to any antibiotic drug prenatally (trimester 1–3) or in the first 2 years of life, among 990,098 births (1996–2012). For prenatal exposure, outcomes after birth until 2014 were considered. For postnatal exposure (first 6 months, 6–11 month after birth, 1–2 years after birth), outcomes after 2 years of age until 2014 were considered. The following factors were adjusted for: maternal age, parity, maternal smoking during pregnancy [yes/no], mother unmarried [yes/no], mother born elsewhere than Finland [yes/no], cesarean section [yes/no], mother’s inpatient care due to mental health disorders [yes/no], mother’s purchase of psychotropic drugs (N05 or N06) during pregnancy [yes/no], mother’s diagnoses related to systemic inflammatory disorders [yes/no], multiple birth [yes/no], offspring sex, perinatal health problems (birth weight < 2500 grams, gestational age < 37 weeks or small for gestational age according to Finnish sex-specific standards) [yes/no]. For postnatal exposure we adjusted also for mother’s prescriptions for antibiotics during pregnancy [yes/no]. Error bars indicate 99% confidence interval. Reference: Births where the fetuses or offspring were not exposed to any antibiotic drug during the exposure period studied.

Antibiotic exposure during pregnancy (T1–T3) associated with a 14–15% increased risk for any offspring psychiatric diagnosis from birth until 2014, after adjusting for pregnancy- and birth-related factors (HR_T1_ = 1.15 [1.13–1.18]; HR_T2_ = 1.14 [1.12–1.16]; HR_T3_ = 1.14 [1.12–1.17], for HR_T1-T3_ see Fig. 1). When assessing F-diagnosis groups separately, the risk increase was strongest for sleep disorders (F51, HR_T1–T3_ = 1.36 [1.25–1.46]), while there was no increased risk for eating disorders (F50) or autism (F84) (Fig. 1). The antibiotic drugs were categorized into (i) airway antibiotics (J01C, D or F), (ii) urinary tract or soft tissue antibiotics (J01E or M), narrow spectrum antibiotics (J01C) and broad spectrum antibiotics (J01A, B, E, F, G, M, R or X; dominated by J01A, E, F and M). The adjusted risk for any psychiatric diagnosis was similarly low for airway antibiotics (HR_T1–T3_ = 1.16 [1.14–1.18]), urinary tract antibiotics (HR_T1–T3_ = 1.20 [1.02–1.40]), after exposure to both (HR_T1–T3_ = 1.18 [1.02–1.36], after narrow spectrum (HR_T1–T3_ = 1.12 [1.10–1.15], and even after broad spectrum antibiotics (HR_T1–T3_ = 1.19 [1.14–1.24], Table 1). When, for these exposures, assessing F-diagnosis groups separately, sample sizes were generally low for the urinary tract antibiotics resulting in wide 99% CIs. However, looking at exposure to the other antibiotic groups (airway, narrow spectrum and broad spectrum antibiotics) revealed that the risk increase was strongest for sleep disorders (F51, point-wise HR_T1–T3_ = 1.32–1.41, Table 1). A robust effect was seen also on mood disorders (F30–39, F92, point-wise HR_T1–T3_ = 1.12–1.41), anxiety disorders (F40-43, F93, point-wise HR_T1–T3_ = 1.13–1.43), ADHD/conduct disorder (F90-91, point-wise HR_T1–T3_ = 1.17–1.53) and on other behavioral and emotional disorders (F98, point-wise HR_T1–T3_ = 1.14–1.29) (Table 1). This was supported by a slightly increased risk for purchase of any psychotropic drug (N05, N06A or N06B) after antibiotic exposure (point-wise HR_T1–T3_ = 1.14–1.17), with highest HR being for antidepressants (N06A, HR = 1.28 [99% CI: 1.15–1.42], Table 1). Broad spectrum compared to narrow spectrum antibiotics implied a slightly higher risk for mood and anxiety disorders, other behavioral and emotional disorders (F98) and purchase of antidepressants (N06A) (Table 1).

**Table 2:** Cox hazard ratios (HR) and 99% confidence intervals (CI) for the outcomes (columns) (i) any psychiatric disorder (ICD-10 F diagnoses), and (ii) any psychotropic medication (ATC N05 and N06) until 2014, in relation to A) maternal purchase of antibiotic drug within 3 months before pregnancy, during pregnancy, and within 3 month after pregnancy, and B) maternal infection and/or purchase of antibiotic drug at least during pregnancy.

Exposures and time period (proportion of births)	Any F diagnosis		Any psychotropic medication	
	HR	99% CI	HR	99% CI
A) Any antibiotic drug (ATC J01)				
Only within 3 months before pregnancy (3 mB)				
No (92.5%)	1.00		1.00	
Yes (7.5%)	1.07	1.03–1.10	1.06	1.01–1.10
Only during pregnancy (P)				
No (84.6%)	1.00		1.00	
Yes (15.4%)	1.16	1.14–1.18	1.16	1.13–1.19
Only within 3 months after pregnancy (3 mA)				
No (86.1%)	1.00		1.00	
Yes (13.9%)	1.02	1.00–1.09	1.03	1.00–1.06
At least during pregnancy				
No (74.3%)	1.00		1.00	
Yes (25.7%)	1.16	1.14–1.18	1.16	1.13–1.19
B) Infection^a^ and/or any antibiotic drug in the same model				
At least during pregnancy				
No (74.3%)	1.00		1.00	
Infection (2.3%)	1.21	1.15–1.28	1.28	1.18–1.38
Any antibiotic drug (ATC J01) (25.7%)	1.15	1.13–1.17	1.16	1.13–1.19

^a^Infections considered included: ICD-10 A00-A99, B00-B99, J00-J22, K35 and L00-L08 and O23

‘No’ were all those not in the corresponding ‘Yes’ category, irrespectively of exposure in another period or not

Adjusted for the variables maternal age, parity, maternal smoking during pregnancy [yes/no], mother unmarried [yes/no], mother born elsewhere than Finland [yes/no], cesarean section [yes/no], mother’s inpatient care due to mental health disorders [yes/no], mother’s purchase of psychotropic drugs (N05 or N06) during pregnancy [yes/no], mother’s diagnoses related to systemic inflammatory disorders [yes/no], multiple birth [yes/no], offspring sex, perinatal health problems (birth weight < 2500 grams, gestational age < 37 weeks or small for gestational age according to Finnish sex-specific standards) [yes/no]

To explore if the modest effect sizes where because of unmeasured familial confounding a sibling pair analysis was performed. Any antibiotic drug (any J01) exposure prenatally for the first child was associated with a slightly increased risk of any F-diagnosis for the unexposed second child, using as reference the risk for the second child in sibling pairs where none of the first or second pregnancies were exposed to any antibiotic drug (HR = 1.06 [95% CI: 1.00–1.13]). When also the second child was exposed prenatally the risk was larger (HR = 1.24 [95% CI: 1.16–1.33]). The risk for any psychotropic medication in the second child was not increased for those where only the first child was exposed (HR = 1.08 [95% CI: 0.98–1.18]). However, when exposure also during the second pregnancy, this second child had an increased risk of any psychotropic medication (HR = 1.22 [95% CI: 1.10–1.36]) (Supplementary Table S3).

**Fig. 2:**
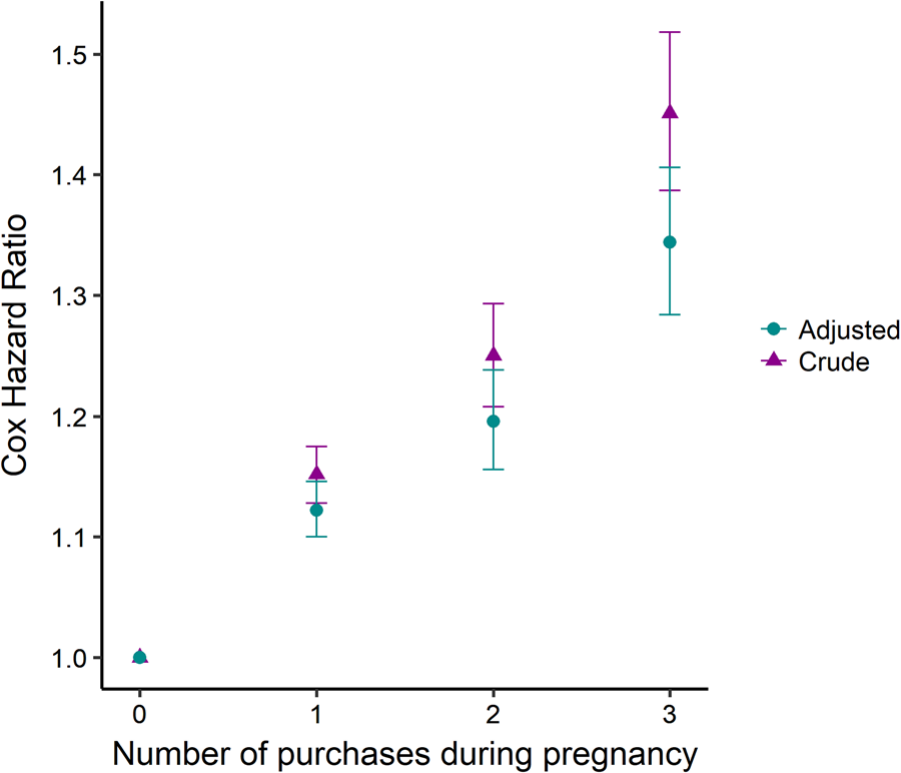
Risk for any offspring psychiatric diagnosis by increasing number of mother’s antibiotic drug purchases during pregnancy (trimester 1–3). All types of antibiotic drugs were considered. The covariates adjusted for are those listed in Fig. 1. Error bars indicate 99% confidence interval. Reference: Births where the mother had no antibiotic purchase during pregnancy.

Moreover, maternal antibiotic drug purchase during the 3 months before (3 mB) or 3 months after (3 mA) pregnancy had smaller risk effect size for any F-diagnosis in offspring (HR_3mB_ = 1.07 [99% CI: 1.03–1.10] and HR_3mA_ = 1.02 [99% CI: 1.00–1.09], respectively) than the purchase during pregnancy (P) HR_P_ = 1.16 [99% CI: 1.14–1.18]. This was supported by lower risk estimates also for offspring N05 and N06 purchases upon maternal exposure before or after pregnancy (HR_3mB_ = 1.06 [99% CI: 1.01–1.10], HR_3mA_ = 1.03 [99% CI: 1.00–1.06] than during pregnancy HR_P_ = 1.16 [99% CI: 1.13–1.19], respectively) Table 2). This together with the sibling pair analysis suggest that the exposure effects detected are not only because of familial confounding. Accordingly, there was a modest dose effect of any antibiotic exposure on any psychiatric disorder (Fig. 2).

Maternal infection during pregnancy implied a higher risk for offspring psychotropic drug purchase than maternal purchase of antibiotic drug did HR_Infection_ = 1.28 [99% CI: 1.18–1.38] HR_Antibiotic drug_ = 1.16 [99% CI: 1.13–1.19] (Table 2). However, adjustment for maternal infection during pregnancy did not change the risk estimates of maternal antibiotic drug purchase on offspring F-diagnosis (HR = 1.16 [99% CI: 1.14–1.18]) or on psychotropic drug purchase (HR = 1.16 [99% CI: 1.13–1.19]) (Table 2).

### Postnatal exposure to antibiotic drugs

The exposure was stratified in the age groups: the first 6 months (proportion_exposed_ = 13.8%), 6–11 months (proportion_exposed_ = 41.1%) and 1–2 years of age (proportion_exposed_ = 53.9%). The adjusted risk for any psychiatric diagnosis after 2 years of age by purchase of any antibiotic drug (any J01) was only slightly increased, irrespective of age at exposure (point-wise HR = 1.10–1.16; Table 3 and Fig. 1). However, the risks of any antibiotic drug for specific psychiatric diagnosis groups were higher. The strongest risk was seen on sleep disorders after exposure during the second year (F51, HR_1-2 years_ = 1.40 [1.24–1.60]), followed by ADHD/conduct disorder (F90–91, HR_1-2 years_ = 1.26 [1.20–1.33]) and other behavioral and emotional disorders (F98, HR_1–2 years_ = 1.24 [1.17–1.31]). Only autism (F84) showed no risk increase for any age group (Table 3 and Fig. 1). Excluding the offspring with an in-patient care for infectious disorder (A00-B99, G00-G03, J00-J22, K35, L08, N10, N30) before 2 years of age (12.3%), because of lack of in-patient antibiotic treatment data, reduced the adjusted HRs slightly for the largest psychiatric diagnosis groups (Supplementary Table S4 Model 2). However, the risk estimates in Model 2 remained significantly above reference indicating that the risk estimates in the original sample (Model 1) were not completely explained by the hospitalized infection cases. Including these same in-patient care offspring with infections in the exposed group resulted in an overall increase of the HRs that was strongest for offspring exposed before 6 months of age (Table S4 Model 3). This latter finding supports an association for early infection or antibiotic drug exposure with later psychiatric disorders. That the HR increase was strongest in those exposed before 6 months of age probably reflects that this group enlarged the most in size in Model 3 compared to Model 2 and Model 1.

**Table 3:** Adjusted Cox hazard ratios (HR) and 99% confidence intervals (CI) for the outcomes (columns) (i) psychiatric diagnoses (ICD-10 F diagnoses), and (ii) psychotropic medication until 2014, in relation to postnatal exposure to antibiotics (rows) in the first 2 years of life, among the 990 098 births (1996-2012). Outcomes after 2 years of age are considered.

Antibiotic drugs	Any F diagnosis		F30-39, F92		F40-43, F93		F50		F51		F84		F90-91		F98		Any psychotropic medication		N05		N06A		N06B	
ATC code groups	*n* = 98519		*n* = 19149		*n* = 27164		*n* = 3320		*n* = 2002		*n* = 7495		*n* = 19706		*n* = 17825		*n* = 56340		*n* = 37491		*n* = 13050		*n* = 16722	
J01CDF or J01EM	HR	99% CI	HR	99% CI	HR	99% CI	HR	99% CI	HR	99% CI	HR	99% CI	HR	99% CI	HR	99% CI	HR	99% CI	HR	99% CI	HR	95% CI	HR	99% CI
First 6 months																								
No *n* = 853177	1.00		1.00		1.00		1.00		1.00		1.00		1.00		1.00		1.00		1.00		1.00		1.00	
Any J01 *n* = 136961	1.15	1.12–1.17	1.17	1.12–1.23	1.15	1.11–1.20	1.11	0.97–1.26	1.11	1.05–1.20	0.99	0.91–1.08	1.16	1.11–1.22	1.13	1.01–1.28	1.25	1.22–1.29	1.31	1.26–1.35	1.16	1.09–1.23	1.22	1.16–1.29
Airway (CDF) *n* = 126537	1.15	1.12–1.17	1.19	1.13–1.25	1.16	1.11–1.21	1.10	0.97–1.26	1.07	0.94–1.24	0.99	0.91–1.08	1.17	1.12–1.23	1.07	1.02–1.14	1.25	1.21–1.29	1.30	1.25–1.34	1.17	1.10–1.25	1.23	1.17–1.30
Urinary tract, soft tissue (EM) *n* = 3235	1.41	1.24–1.61	1.10	0.75–1.61	1.34	1.00–1.78	1.43	0.61–3.38	1.70	0.92–3.23	1.40	0.88–2.23	1.32	0.97–1.80	1.31	1.01–1.66	1.57	1.31–1.88	1.76	1.43–2.16	1.26	0.80–1.97	1.19	0.83–1.71
CDF and EM^a^ *n* = 7149	1.39	1.27–1.53	1.21	0.92–1.60	1.45	1.18–1.78	1.64	0.87–3.06	1.40	0.83–2.30	0.86	0.57–1.30	1.27	1.01–1.59	1.34	1.13–1.60	1.68	1.48–1.92	1.88	1.62–2.19	1.13	0.78–1.63	1.36	1.06–1.73
6–11 months																								
No *n* = 583036	1.00		1.00		1.00		1.00		1.00		1.00		1.00		1.00		1.00		1.00		1.00		1.00	
All J01 *n* = 407062	1.10	1.08–1.11	1.10	1.06-1.14	1.10	1.06–1.13	1.06	0.97–1.16	1.09	1.03–1.18	0.90	0.84–0.95	1.12	1.08–1.16	1.20	1.08–1.32	1.17	1.14–1.19	1.22	1.18–1.25	1.06	1.02–1.11	1.16	1.12–1.21
Airway (CDF) *n* = 357981	1.10	1.08–1.12	1.11	1.07–1.15	1.10	1.07–1.14	1.06	0.96-1.16	1.10	0.96–1.22	0.90	0.85–0.96	1.13	1.09–1.17	1.05	0.99–1.10	1.16	1.13–1.18	1.19	1.16–1.23	1.08	1.03–1.13	1.17	1.12–1.22
Urinary tract, soft tissue (EM) *n* = 6780	1.19	1.07–1.31	1.11	0.86–1.43	1.08	0.87–1.34	0.85	0.43–1.69	1.54	0.95–2.50	1.20	0.85–1.69	1.31	1.05–1.63	0.92	0.73–1.15	1.42	1.25–1.61	1.55	1.34–1.80	1.06	0.78–1.44	1.31	1.03–1.68
CDF and EM^a^ *n* = 42301	1.21	1.16–1.26	1.19	1.07–1.33	1.22	1.12–1.34	1.36	1.05–1.76	1.50	1.21–1.90	0.83	0.70–0.98	1.20	1.09–1.32	1.07	0.97–1.20	1.46	1.38–1.54	1.63	1.53–1.74	1.01	0.88–1.17	1.13	1.13–1.40
1–2 years																								
No *n* = 456012	1.00		1.00		1.00		1.00		1.00		1.00		1.00		1.00		1.00		1.00		1.00		1.00	
All J01 *n* = 534086	1.16	1.13–1.18	1.18	1.12–1.23	1.16	1.11–1.21	1.22	1.09–1.37	1.40	1.24–1.60	0.96	0.89–1.03	1.26	1.20–1.33	1.24	1.17–1.31	1.40	1.36–1.44	1.53	1.47–1.58	1.17	1.11–1.24	1.31	1.24–1.38
Airway (CDF) *n* = 387396	1.15	1.12–1.17	1.18	1.13-1.24	1.16	1.11–1.21	1.21	1.08–1.36	1.13	0.92–1.41	0.95	0.88–1.02	1.25	1.19–1.31	1.23	1.11–1.34	1.36	1.32–1.40	1.46	1.41–1.52	1.18	1.11–1.25	1.28	1.22–1.36
Urinary tract, soft tissue (EM) *n* = 6694	1.14	1.03–1.26	1.05	0.82–1.36	1.00	0.81–1.24	1.29	0.75–2.23	0.72	0.30–1.48	0.89	0.60–1.32	1.27	1.01–1.62	1.48	1.22–1.80	1.31	1.14–1.51	1.45	1.23–1.72	1.05	0.78–1.42	1.17	0.89–1.54
CDF and EM^a^ *n* = 139996	1.27	1.24–1.31	1.23	1.15–1.32	1.26	1.19–1.33	1.35	1.15–1.59	1.48	1.17–1.85	1.03	0.93–1.13	1.45	1.36–1.54	1.35	1.22–1.50	1.72	1.65–1.78	1.94	1.86–2.04	1.22	1.12–1.33	1.54	1.43–1.65

Any F diagnosis was found for 9.9% of the cohort and included all ICD-10 F diagnoses

ICD-10 F30-39, F92 (1.9%) Mood disorders; F40-43, F93 (2.7%) Anxiety disorders; F50 (0.34%) Eating disorders; F51 (0.51%) Nonorganic sleep disorders; F84 (0.76%) Autism Spectrum Disorder (ASD); F90-91 (2.0%) Attention Deficit Hyperactivity Disorder (ADHD) and Conduct disorders; F98 (2.2%) Other behavioral and emotional disorders with onset usually occurring in childhood and adolescence

Psychotropic medications studied defined according to the Anatomic Therapeutic Chemical (ATC) classification system were found for 5.7% of the cohort and included the following: ATC groups N05 (3.8%) antipsychotics, anxiolytics, hypnotics and sedatives; ATC group N06A (1.3%) antidepressants; ATC group N06B (1.7%) stimulants

Antibiotic drugs (ATC codes):

CDF: Any of J01C, D and/or F, i.e., airway antibiotics against primarily gram-positive bacteria, and no J01EM. EM: Any of J01E and/or M, i.e., urinary tract and soft tissue antibiotics against both gram-positive and gram-negative bacteria, and no J01CDF. J01C: Phenoxymethylpenicillin and penicillins with extended spectrum; J01D: Cephalosporins; J01F: Macrolides; J01E: Sulfonamides and trimethoprim; J01M: Fluoroquinolones

Less than 0.2% of the offspring were treated with any of J01A, J01B, J01G, J01R or J01X, and these exposures were not included in this Table.

^a^Exposure to at least two antibiotic drugs

The models were adjusted for: maternal age, parity, maternal smoking during pregnancy [yes/no], mother unmarried [yes/no], mother born elsewhere than Finland [yes/no], cesarean section [yes/no], mother’s inpatient care due to mental health disorders [yes/no], mother’s purchase of psychotropic drugs (N05 or N06) during pregnancy [yes/no], mother’s diagnoses related to systemic inflammatory disorders [yes/no], multiple birth [yes/no], offspring sex, perinatal health problems (birth weight < 2500 grams, gestational age < 37 weeks or small for gestational age according to Finnish sex-specific standards) [yes/no], and mother’s prescriptions for antibiotics during pregnancy [yes/no]

Stratifying for type of antibiotic drug, the strongest risk for any psychiatric diagnosis (Any F diagnosis) after 2 years of age was seen for the offspring exposed before 6 months of age to urinary tract and soft tissue antibiotics with or without combination with airway antibiotics (HR~1.4) (Table 3). Consistent between most exposure categories (defined by antibiotic drug group and exposure age), increased risks were seen for ADHD/conduct disorder (F90–91, point-wise HR = 1.12–1.45), anxiety disorders (F40–43 and F93, point-wise HR = 1.10–1.45), other behavioral and emotional disorders (F98, point-wise HR = 1.07–1.48), and, albeit weaker, for mood disorders (F30–39 and F92, point-wise HR = 1.10–1.23), while the risk was significantly increased for sleep disorders only after exposure to the ‘both’ antibiotic group (airway and urinary tract antibiotics) after 6 months of age (F51, point-wise HR = 1.48–1.50).

The HRs for any psychotropic medication purchase to offspring after two years of age were stronger (point-wise HR: 1.17–1.40) than the corresponding HRs for any psychiatric diagnosis (Fig. 1). High risk estimates were seen primarily for purchase of N05 medication (point-wise HR: 1.19–1.94, Table 3), in this cohort corresponding primarily to anxiolytics (N05B) and sedatives (N05C) used for treatment of anxieties and sleep problems. Further, there was a robustly increased risk for purchase of stimulants for most exposure categories (N06B, point-wise HR: 1.13–1.54) which are used in the treatment of ADHD. Dose effects were detected among children exposed during their second year comparing the ‘CDF and EM’ antibiotic category corresponding to at least two drugs, with the ‘EM’ category for any psychiatric diagnosis and any psychotropic medication (Table 3).

## Discussion

Our findings indicate that exposure to antibiotic drugs, in utero and the first two postnatal years, is associated with a modestly increased risk for pediatric psychiatric disorders, including sleep disorders, ADHD and conduct disorder, mood and anxiety disorders, as well as the diagnosis group of other behavioral and emotional disorders with onset usually occurring in childhood and adolescence. The associations between antibiotic drug exposure and later psychiatric disorders might reflect direct effects of the targeted infections, as well as effects of antibiotic drugs on barrier microbiota with downstream opportunistic infections and altered gut-brain axis signaling. Specifically, fetal exposure was associated with a 32–41% (point-wise HR = 1.32–1.41) increased risk for sleep disorders spanning different types of antibiotics, whereas the risk estimates for ADHD/conduct disorder, mood, anxiety and other behavioral and emotional disorders increased with 12–53%. To strengthen these findings we estimated the risk for psychotropic medication given exposure to the antibiotic drugs. The elevated risk for mood disorders was supported by a 28% increased risk for antidepressant purchase after broad spectrum antibiotics exposure. Other support of an association between any prenatal antibiotic exposure and offspring psychiatric disorder included the dose–response effect observed illustrated in Fig. 2, the lower effect size of the association between maternal antibiotic use outside compared to during pregnancy, and finally the sibling pair analysis showing that, among singleton sibling pairs, the second child had a stronger increased risk for any F diagnosis or any psychotropic medication when it was exposed compared to when only its older sibling was exposed. Antibiotic exposure during the first two postnatal years showed similar modest effects as fetal exposure did on the aforementioned psychiatric diagnosis groups. Thus, there was no robust effect size difference between exposure prenatally, the first 6 month, the next 6 months or the second year on risk for any later psychiatric diagnosis. Notably however, after postnatal exposure the risks for later psychotropic medication were somewhat stronger than that for psychiatric diagnoses. Higher risk estimates were seen primarily for purchase of anxiolytic and sedative (N05) medication (19–94% elevated risk), used for treatment of anxieties and sleep problems. Further, there was a robustly increased risk for purchase of stimulants (N06B, 13–54% increased risk), which are used in the treatment of ADHD. Type of antibiotics, grouped into airway versus urinary tract and soft tissue antibiotics, had no robust influence on the effect of prenatal exposure, but broad spectrum antibiotics implied a slightly higher risk than narrow spectrum antibiotics for mood and anxiety disorders, other behavioral and emotional disorders, and purchase of antidepressants. For postnatal exposure, stronger effects on any psychiatric diagnosis and any psychotropic medication were detected among offspring exposed to urinary tract and soft tissue antibiotics, which often are broad spectrum, compared to the more commonly narrow spectrum airway antibiotics. However, when it comes to specific psychiatric diagnosis groups, due to small exposure groups for urinary tract and soft tissue antibiotics, differences in effects between antibiotic types could not be detected but cannot be ruled out.

Previous nation-wide longitudinal register studies on childhood infection and anti-infective drug exposure and later mental disorders have been performed primarily in Denmark. Köhler et al. found increased risks (HR = 1.3–1.7) for schizophrenia and mood disorders, in their study of antibiotic drug exposure from 10 to 28 years of age, while smaller or no effects were seen of other anti-infective drug^7^. They also followed all births from 1995 to 2013 and reported increased risks for several psychiatric disorders after infections and anti-infective drug exposure, especially for those treated with antibiotics (HR for any psychiatric diagnosis = 1.4–1.5) and infections requiring hospitalization (HR = 1.7–2.0)^8^. However, mental disorders were grouped differently than in our study prohibiting result comparison, e.g. sleeping disorders and ADHD/conduct disorders were not studied separately and both mood and anxiety disorders were split into several diagnosis groups. Finally, Lydholm et al. recently suggested that the associations of prenatal exposure to anti-infectives and infection with any offspring pediatric psychiatric diagnosis were explained by unmeasured factors, based on comparing risk estimates from maternal exposure during pregnancy with that of exposure before and after pregnancy, and comparing risks from maternal versus paternal exposures^15^. However, we found an increased risk estimate by exposure specifically during pregnancy. That we but not Lydholm et al.^15^ detected pregnancy specific effect might be because we studied specifically antibiotics, which have previously been reported to be associated with larger risk estimates than other anti-infectives at postnatal exposure^7^. In Canadian register studies investigating effects of antibiotic exposure during the first one-two years of life on risk for autism or ADHD no associations were found^17,18^, the former in agreement with our data.

Most current nation-wide register data (e.g. the Finnish used here) are not reliable enough to determine whether the associations between antibiotic drug exposure and later psychiatric disorders reflect the targeted infections, or antibiotic drug-induced barrier dysmicrobiota with secondary opportunistic infections and/or possibly alterations in gut-brain axis signaling. This is primarily because less severe infections are under-reported, antibiotic drugs are overprescribed^44,45^, and compliance to these prescriptions is not complete^46^. The extent of these factors likely vary between countries. In our study, all recorded drugs were not only prescribed, but also purchased. Further, in our data maternal infection during pregnancy did increase the risk for offspring psychotropic drug purchase more than maternal purchase of any antibiotic drug did, but the risk estimates of the latter exposure were not affected by adjustment for the registered maternal infection. Because of considerable under-reporting of infection in our data we adjusted for maternal infections only in the aforementioned small sensitivity analysis. For the first two postnatal years, we had infection data only for certain hospitalized bacterial infections. However, these cases did not completely explain the risk estimates of antibiotic drug purchase on psychiatric disorder. Our tendencies for stronger effects of antibiotics with a broader target spectrum might reflect more severe infections, as well as larger effects on gut microbiota. Both infections and a dysfunctional gut microbiota can influence the immune system. The evidence for a proinflammatory state in pediatric population is strongest for ASD, but proinflammatory cytokines have been reported elevated in children with major depressive disorder, bipolar disorder, post-traumatic stress disorder, obsessive-compulsive disorder, Tourette’s disorder, ADHD and schizophrenia^9^. In adults there is support for immune activation in the pathophysiology of major depressive disorder^47^, and schizophrenia^48^. Immunomodulation by the gut microbiota is emerging as an important pathway that orchestrates the microbiota–gut–brain communication, and thereby it may affect neurodevelopment^10,49–51^. Further, reduction of gram-negative bacteria in the mouse gut microbiota, e.g., by exposure to antibiotics, caused inadequate regulation of circadian clocks in the intestinal epithelial cells, which has negative downstream effects on lipid metabolism^52^, and potentially on brain housekeeping functions^53^ and, as we hypothesize here, on sleep as well.

Other limitations include that the sample size of later onset psychiatric disorders was reduced as only the oldest birth year cohort was followed up for 18 years. We estimated the proportion identified in the complete cohort out of the number of cases predicted to be diagnosed before 19 years of age using the diagnosis rates in the 1996 birth cohort, thus not considering general changes over time in incidence of the diagnoses. Twenty-nine percent of eating disorders, 33% of mood disorders, 41% of anxiety disorders, and 60–85% of sleeping disorders, ASD, ADHD and conduct disorders, and other behavioral and emotional disorders (F98) were identified. This was in agreement with the data on mean age at onset of the diagnosis groups (Table S1). The risk estimates for offspring psychiatric diagnosis or medication did not follow the pattern of proportion picked up or mean age at onset. Second, the aim of this study was to explore effect of antibiotic exposure during fetal and the first 2 years of life. A lot can happen during the years between exposure and outcome, partly depending on outcome onset time (Table S1). However, adjustment in the fetal exposure model for exposure during the first 2 years did not change the risk estimates. We did not account for other treatment-confounder effects such as the influence of exposure to antibiotics after the second year birthday since its effect on the risk of any psychiatric diagnosis declined by increasing exposure age, and the vast majority (83.2%) of the full cohort was exposed to antibiotic drug up to the age of 18 years. Third, the validity of psychiatric diagnoses other than autism of the pediatric population in HILMO has not been reported. Fourth, early psychosocial adversity increases the risk for later psychopathology^54,55^. We adjusted for presence of maternal in-patient psychiatric diagnoses and psychotropic medication, and the sibling pair analysis, as well as analysis of maternal exposure outside the pregnancy period proposed that the effects where not completely explained by familial confounding. Still, the effect sizes reported should be considered to possibly be influenced by residual confounding from genetic and environmental influences related to paternal psychiatric conditions that could influence medical care seeking not possible to adjust for here. The estimated risk needed for such unmeasured confounding, conditional the measured covariates, to explain away the associations reported here would be at least HR = 2.1 [99% CI: 1.6–2.8] for prenatal exposure and HR = 3.3 [3.1–3.5] for postnatal exposure, towards both the exposure and the outcome^56^. Finally, the associative nature of the study prevents causal inference.

## Conclusion

There is a modest association between early life antibiotic exposure and later childhood development of sleep disorders, ADHD, conduct disorder, mood and anxiety disorders, and other behavioral and emotional disorders. The magnitudes of the associations overlap considerably between diagnoses. Given the high occurrence of early-life antibiotic exposure, and the substantial prevalence of childhood- and adolescent-onset psychopathology, modest associations among these phenomena are of public health relevance. However, larger studies are needed to confirm the effect sizes of the individual diagnoses groups after controlling for confounding by family factors, and to explore whether the associations detected reflect effects of the antibiotic drug use or of the targeted infections.

## Supplementary information

Supplementary data

## References

[1] Benros ME (2013). Autoimmune diseases and severe infections as risk factors for mood disorders: a nationwide study. JAMA Psychiatry.

[2] Khandaker GM, Dantzer R, Jones PB (2017). Immunopsychiatry: important facts. Psychol. Med..

[3] Estes ML, McAllister AK (2016). Maternal immune activation: Implications for neuropsychiatric disorders. Science.

[4] Yolken R (2016). Individuals hospitalized with acute mania have increased exposure to antimicrobial medications. Bipolar Disord..

[5] Betts KS, Salom CL, Williams GM, Najman JM, Alati R (2015). Associations between self-reported symptoms of prenatal maternal infection and post-traumatic stress disorder in offspring: evidence from a prospective birth cohort study. J. Affect Disord..

[6] Adam Y, Meinlschmidt G, Lieb R (2013). Associations between mental disorders and the common cold in adults: a population-based cross-sectional study. J. Psychosom. Res..

[7] Kohler-Forsberg O (2019). A Nationwide Study in Denmark of the association between treated infections and the subsequent risk of treated mental disorders in children and adolescents. JAMA Psychiatry.

[8] Kohler O (2017). Infections and exposure to anti-infective agents and the risk of severe mental disorders: a nationwide study. Acta Psychiatr. Scand..

[9] Mitchell RH, Goldstein BI (2014). Inflammation in children and adolescents with neuropsychiatric disorders: a systematic review. J. Am. Acad. Child Adolesc. Psychiatry.

[10] Fung TC, Olson CA, Hsiao EY (2017). Interactions between the microbiota, immune and nervous systems in health and disease. Nat. Neurosci..

[11] Rice D, Barone S (2000). Critical periods of vulnerability for the developing nervous system: evidence from humans and animal models. Environ. Health Perspect..

[12] Blomstrom A (2016). Associations between maternal infection during pregnancy, childhood infections, and the risk of subsequent psychotic disorder–A Swedish Cohort Study of Nearly 2 million individuals. Schizophr. Bull..

[13] Dreier JW (2018). Fever and infections during pregnancy and psychosis-like experiences in the offspring at age 11. A prospective study within the Danish National Birth Cohort. Psychol. Med..

[14] Flinkkila E, Keski-Rahkonen A, Marttunen M, Raevuori A (2016). Prenatal inflammation, infections and mental disorders. Psychopathology.

[15] Lydholm CN (2019). Parental infections before, during, and after pregnancy as risk factors for mental disorders in childhood and adolescence: A Nationwide Danish Study. Biol. Psychiatry.

[16] Slykerman RF (2017). Antibiotics in the first year of life and subsequent neurocognitive outcomes. Acta Paediatr..

[17] Hamad AF, Alessi-Severini S, Mahmud SM, Brownell M, Kuo IF (2018). Early childhood antibiotics use and autism spectrum disorders: a population-based cohort study. Int J. Epidemiol..

[18] Axelsson PB (2019). Investigating the effects of cesarean delivery and antibiotic use in early childhood on risk of later attention deficit hyperactivity disorder. J. Child Psychol. Psychiatry.

[19] Guida F (2018). Antibiotic-induced microbiota perturbation causes gut endocannabinoidome changes, hippocampal neuroglial reorganization and depression in mice. Brain Behav. Immun..

[20] O’Mahony SM (2014). Disturbance of the gut microbiota in early-life selectively affects visceral pain in adulthood without impacting cognitive or anxiety-related behaviors in male rats. Neuroscience.

[21] Desbonnet L (2015). Gut microbiota depletion from early adolescence in mice: Implications for brain and behaviour. Brain Behav. Immun..

[22] O’Mahony SM, Clarke G, Dinan TG, Cryan JF (2017). Early-life adversity and brain development: Is the microbiome a missing piece of the puzzle?. Neuroscience.

[23] Mueller NT, Bakacs E, Combellick J, Grigoryan Z, Dominguez-Bello MG (2015). The infant microbiome development: mom matters. Trends Mol. Med..

[24] Clarke G, O’Mahony SM, Dinan TG, Cryan JF (2014). Priming for health: gut microbiota acquired in early life regulates physiology, brain and behaviour. Acta Paediatr..

[25] Rodriguez JM (2015). The composition of the gut microbiota throughout life, with an emphasis on early life. Micro. Ecol. Health Dis..

[26] Borre YE (2014). Microbiota and neurodevelopmental windows: implications for brain disorders. Trends Mol. Med..

[27] Fouhy F (2012). High-throughput sequencing reveals the incomplete, short-term recovery of infant gut microbiota following parenteral antibiotic treatment with ampicillin and gentamicin. Antimicrob. Agents Chemother..

[28] Johnson CL, Versalovic J (2012). The human microbiome and its potential importance to pediatrics. Pediatrics.

[29] Hussey, S., et al. Parenteral antibiotics reduce bifidobacteria colonization and diversity in neonates. *Int. J. Microbiol*. **2011**, 130574 (2011). 10.1155/2011/130574 PMC2929493 20811542

[30] Artama M, Gissler M, Malm H, Ritvanen A (2011). Drugs, Pregnancy Study G. Nationwide register-based surveillance system on drugs and pregnancy in Finland 1996–2006. Pharmacoepidemiol. Drug Saf..

[31] Gissler M, Teperi J, Hemminki E, Merilainen J (1995). Data quality after restructuring a national medical registry. Scand. J. Soc. Med..

[32] Furu K (2010). The Nordic countries as a cohort for pharmacoepidemiological research. Basic Clin. Pharm. Toxicol..

[33] Klaukka T (2001). The Finnish database on drug utilization. Norwegian J. Epidemiol..

[34] Sund R (2012). Quality of the finnish hospital discharge register: a systematic review. Scand. J. Public Health.

[35] Lampi KM (2010). Brief report: validity of Finnish registry-based diagnoses of autism with the ADI-R. Acta Paediatr..

[36] Almqvist C (2015). Individual maternal and child exposure to antibiotics in hospital - a national population-based validation study. Acta Paediatr..

[37] Sankilampi U, Hannila ML, Saari A, Gissler M, Dunkel L (2013). New population-based references for birth weight, length, and head circumference in singletons and twins from 23 to 43 gestation weeks. Ann. Med..

[38] Clayton PE (2007). Management of the child born small for gestational age through to adulthood: a consensus statement of the International Societies of Pediatric Endocrinology and the Growth Hormone Research Society. J. Clin. Endocrinol. Metab..

[39] Gissler M, Merilainen J, Vuori E, Hemminki E (2003). Register based monitoring shows decreasing socioeconomic differences in Finnish perinatal health. J. Epidemiol. Community Health.

[40] Class QA, Rickert ME, Larsson H, Lichtenstein P, D’Onofrio BM (2014). Fetal growth and psychiatric and socioeconomic problems: population-based sibling comparison. Br. J. Psychiatry.

[41] Chang Z (2014). Maternal age at childbirth and risk for ADHD in offspring: a population-based cohort study. Int J. Epidemiol..

[42] Ekblad M, Gissler M, Lehtonen L, Korkeila J (2010). Prenatal smoking exposure and the risk of psychiatric morbidity into young adulthood. Arch. Gen. Psychiatry.

[43] van der Burg JW (2016). The role of systemic inflammation linking maternal BMI to neurodevelopment in children. Pediatr. Res..

[44] Lucas PJ, Cabral C, Hay AD, Horwood J (2015). A systematic review of parent and clinician views and perceptions that influence prescribing decisions in relation to acute childhood infections in primary care. Scand. J. Prim. Health Care.

[45] Dekker AR, Verheij TJ, van der Velden AW (2015). Inappropriate antibiotic prescription for respiratory tract indications: most prominent in adult patients. Fam. Pract..

[46] Kardas P, Devine S, Golembesky A, Roberts C (2005). A systematic review and meta-analysis of misuse of antibiotic therapies in the community. Int J. Antimicrob. Agents.

[47] Dantzer R, O’Connor JC, Freund GG, Johnson RW, Kelley KW (2008). From inflammation to sickness and depression: when the immune system subjugates the brain. Nat. Rev. Neurosci..

[48] Schwarcz R, Bruno JP, Muchowski PJ, Wu HQ (2012). Kynurenines in the mammalian brain: when physiology meets pathology. Nat. Rev. Neurosci..

[49] Rothhammer V (2016). Type I interferons and microbial metabolites of tryptophan modulate astrocyte activity and central nervous system inflammation via the aryl hydrocarbon receptor. Nat. Med..

[50] Braniste V (2014). The gut microbiota influences blood-brain barrier permeability in mice. Sci. Transl. Med..

[51] Erny D (2015). Host microbiota constantly control maturation and function of microglia in the CNS. Nat. Neurosci..

[52] Wang Y (2017). The intestinal microbiota regulates body composition through NFIL3 and the circadian clock. Science.

[53] Musiek ES, Holtzman DM (2016). Mechanisms linking circadian clocks, sleep, and neurodegeneration. Science.

[54] Ostergaard SD, Larsen JT, Petersen L, Smith GD, Agerbo E (2019). Psychosocial adversity in infancy and mortality rates in childhood and adolescence: a birth cohort study of 1.5 million individuals. Epidemiology.

[55] Ostergaard SD (2016). Predicting ADHD by assessment of rutter’s indicators of adversity in infancy. PLoS ONE.

[56] VanderWeele TJ, Ding P (2017). Sensitivity analysis in observational research: introducing the E-Value. Ann. Intern. Med..

