# Supplementary material for: Early exposure to antibiotic drugs and risk for psychiatric disorders: a population-based study
Source: Transl Psychiatry. 2019 Nov 26;9:317. doi: 10.1038/s41398-019-0653-9 (PMC6879739; doi:10.1038/s41398-019-0653-9)
Supplement: Supplementary file 1 — Supplementary data [file 41398_2019_653_MOESM1_ESM.docx]

**Supplementary Data for**

**Early exposure to antibiotic drugs and risk for psychiatric disorders: a population-based study.**

Catharina Lavebratt^1,2^ PhD, Liu L. Yang^1,2^ MD, MaiBritt Giacobini^1,3^ PhD, Yvonne Forsell^4^ PhD, Martin Schalling^1,2^ PhD, Timo Partonen^5^ PhD, Mika Gissler^5,6,7^ PhD

**Supplementary Table S1** ICD-10 codes and corresponding psychiatric disorders and average age at onset considering diagnoses after birth until 2014 (median, 25^th^ and 75^th^ percentiles).

| **ICD-10 codes** | **Psychiatric disorders** | **Proportion identified of the cases predicted to be diagnosed before 19 years of age*** | **Age at onset** | | **Age at**  **onset** | |
| --- | --- | --- | --- | --- | --- | --- |
|  |  |  | **Birth cohort 1996** | | **Birth cohort 1996-2012** | |
|  |  |  | **Median** | **25^th^-75^th^ percentile** | **Median** | **25^th^-75^th^ percentile** |
| F30-39, F92 | Mood disorders | 33.0% | 14.4 | 10.6-16.2 | 11.0 | 7.5-14.4 |
| F40-43, F93 | Anxiety disorders | 41.0% | 14.4 | 10.7-16.3 | 10.0 | 6.3-14.0 |
| F50 | Eating disorders | 28.7% | 14.8 | 13.0-16.2 | 13.4 | 9.0-15.0 |
| F51 | Sleeping disorders | 62.4% | 2.8 | 0.8-14.5 | 1.1 | 0.7-4.4 |
| F84 | Autism spectrum disorder (ASD) | 64.4% | 6.3 | 3.8-10.7 | 5.2 | 3.4-8.1 |
| F90–91 | Attention deficit hyperactivity disorder (ADHD) and conduct disorders | 61.2% | 8.7 | 6.0-13.2 | 7.3 | 5.4-9.6 |
| F98 | Other behavioral and emotional disorders | 85.6% | 6.7 | 5.0-10.0 | 5.6 | 2.7-7.7 |

Abbreviations: ICD-10, International Statistical Classification of Diseases and Related Health Problems, 10th Revision.

*Proportion identified of the number of cases predicted to be diagnosed before 19 years of age. This was estimated as 16 times the number of cases in birth cohort 1996, which was followed up for 18 years.

**Supplementary Table S2** Demographic characteristics of offspring and their mothers. N=990 098 (1996-2012).

| **Offspring variables** | **%** | **Maternal variables** | **%** |
| --- | --- | --- | --- |
| **Offspring sex** |  | **Maternal smoking** |  |
| Boy | 51.1% | No | 82.5% |
| Girl | 48.9% | Stopped in 1st trimester | 3.4% |
| **Number of fetuses** |  | Continued | 11.5% |
| 1 | 97.0% | Missing | 2.6% |
| 2 | 2.9% | **Maternal age** **(years)** |  |
| 3 | 0.1% | <20 | 2.6% |
| **Perinatal problem*** |  | 20–24 | 16.1% |
| Yes | 12.9% | 25–29 | 31.9% |
| No | 87.1% | 30–34 | 31.0% |
| **Exposure during pregnancy** |  | 35–55 | 18.4% |
| All J01 | 25.7% | **Parity** |  |
| 3 months before | 12.6% | 0 | 41.4% |
| 1^st^ trimester (T1) | 9.7% | 1 | 33.5% |
| 2^nd^ trimester (T2) | 11.7% | 2 | 15.3% |
| 3^rd^ trimester (T3) | 9.9% | 3 | 5.3% |
| 3 months after | 20.8% | 4 or more | 4.5% |
| J01A | 0.6% | Missing | 0.1% |
| J01C | 16.2% | **Mother’s marital status** |  |
| J01D | 10.0% | Married | 59.8% |
| J01E | 0.2% | Cohabiting | 28.5% |
| J01F | 3.0% | Other | 9.6% |
| J01M | 0.2% | Missing | 2.1% |
| **Inpatient care in childhood** |  | **Country of birth** |  |
| A00-B99 | 7.8% | Finland | 92.8% |
| J00-J22 | 11.4% | Other | 7.2% |
| G00-G03, K35, L08, N10, N30 | 2.2% | **Mother’s psychiatric diagnoses**** |  |
| Any | 18.7% | No | 98.1% |
| **Mode of delivery** |  | Yes | 1.9% |
| Vaginal | 75.8% | **Mother’s psychotropic medication during pregnancy N05-N06†** |  |
| Instrumental | 7.3% | No | 95.8% |
| Planned CS | 7.2% | Yes | 4.2% |
| Other CS | 9.5% | **Mother's systemic inflammatory diagnoses M30-M36††** |  |
| Missing | 0.1% | No | 98.9% |
|  |  | Yes | 1.1% |

*Perinatal problem: birth before gestational week 37 or birth weight <2500 g, or small for gestational age (SGA) being a birth weight and/or length more than 2 standard deviations (SD) below the sex- and gestational age-specific reference mean (Sankilampi et al., 2013), according to the International Societies of Pediatric Endocrinology and the Growth Hormone Research Society (Clayton et al, 2007).

Abbreviations: CS: caesarean section; T1-T3: pregnancy trimesters.

Antibiotic drugs (Anatomic Therapeutic Chemical [ATC] codes):

(i) Against both gram-positive and gram-negative bacteria: J01A: Tetracyclines.

(ii) Airway antibiotics against primarily gram-positive bacteria: J01C: Phenoxymethylpenicillin and penicillins with extended spectrum; J01D: Cephalosporins; J01F: Macrolides.

(iii) Against urinary tract and soft tissue infections ie both gram-positive and gram-negative bacteria: J01E: Sulphonamides and trimethoprim; J01M: Fluoroquinolones.

J01B, J01G, J01R and J01X are included in ‘All J01’ but the frequences were too low to report individually.

ICD10 A00-B99 and J00-J22: various infections; G00-G03: meningitis; K35: appendicities; L08: skin infections; N10: pyelonephritis; N30: cystitis.

** Pre-pregnancy in-patient care records: ICD–8: 290–317 in 1969–1986, ICD–9: 290–319 in 1987–1995, and ICD–10: F00–F99 in 1996–2014. †ATC N05 or N06 (antipsychotics, anxiolytics, hypnotics, sedatives, antidepressants, stimulants, nootropics). †† Pre-pregnancy ICD-10 M30-M36.

**References**:

Sankilampi U, Hannila ML, Saari A, Gissler M, Dunkel L. New population-based references for birth weight, length, and head circumference in singletons and twins from 23 to 43 gestation weeks. *Annals of medicine*. 2013;45(5-6):446-454.

Clayton PE, Cianfarani S, Czernichow P, Johannsson G, Rapaport R, Rogol A. Management of the child born small for gestational age through to adulthood: a consensus statement of the International Societies of Pediatric Endocrinology and the Growth Hormone Research Society. *The Journal of clinical endocrinology and metabolism*. 2007;92(3):804-810.

**Supplementary Table S3** Sibling pair analysis in all mothers with a singleton sibling pair among the 990 098 births (birth years 1996-2012). Adjusted Cox hazard ratios (HR) and 95% confidence intervals (CI) for the outcomes (columns) in the second child being (i) any psychiatric disorder (ICD-10 F diagnoses), and (ii) any psychotropic medication (ATC N05 and N06) from birth until 2014, in relation to exposure for the sibling pair to antibiotics in fetal life, trimesters 1-3.

| **Exposure to any antibiotics drug (ATC J01)** | **Any F diagnosis** | | **Any psychotropic medication** | |
| --- | --- | --- | --- | --- |
|  | HR | 95% CI | HR | 95% CI |
| **Model 1** |  |  |  |  |
| None of the siblings in the pair was exposed | 1.00 |  | 1.00 |  |
| Exposure in the first, not in the second pregnancy/child | 1.09 | 1.02-1.16 | 1.09 | 0.99-1.20 |
| Both of the siblings in the pair were exposed | 1.29 | 1.21-1.38 | 1.24 | 1.12-1.38 |
| **Model 2** |  |  |  |  |
| None of the siblings in the pair was exposed | 1.00 |  | 1.00 |  |
| Exposure in the first, not in the second pregnancy/child | 1.06 | 1.00-1.13 | 1.08 | 0.98-1.18 |
| Both of the siblings in the pair were exposed | 1.24 | 1.16-1.33 | 1.22 | 1.10-1.36 |

Birth-related factors adjusted for:

**Model 1**: Adjusted for the variables maternal age, parity, maternal smoking during pregnancy [yes/no], mother unmarried [yes/no], mother born elsewhere than Finland [yes/no], caesarean section [yes/no], mother’s inpatient care due to mental health disorders [yes/no], mother’s purchase of psychotropic drugs (N05 or N06) during pregnancy [yes/no], mother’s diagnoses related to systemic inflammatory disorders [yes/no], multiple birth [yes/no], offspring sex, perinatal health problems (birth weight < 2500 grams, gestational age < 37 weeks or small for gestational age according to Finnish sex-specific standards) [yes/no], and intra-pregnancy interval.

**Model 2**: Adjusted for the variables in Model 1 and any F diagnosis or psychotropic medication, respectively, in the first child.

**Supplementary Table S4** Sensitivity analyses considering in-patient diagnosis for infection before 2 years of age (A00-B99, G00-G03, J00-J22, K35, L08, N10, N30). Exposure: All J01 antibiotic drugs in the first 2 years of life. Adjusted Cox hazard ratios (HR) and 99% confidence intervals (CI) for the outcomes (columns) (i) psychiatric diagnoses (ICD-10 F diagnoses), and (ii) psychotropic medication from birth, in relation to postnatal exposure to antibiotics (rows), among the births (1996-2012) (Models 1 and 3: 990 098 births; Model 2: n=805 431 births).

| **Age at exposure** | **Any F diagnosis** | | **F30-39,**  **F92** | | | **F40-43,**  **F93** | | | **F50** | | | **F51** | | | **F84** | | **F90-91** | | **F98** | | | **Any psychotropicmedication** | | **N05** | | | **N06A** | | | **N06B** | | |  |
| --- | --- | --- | --- | --- | --- | --- | --- | --- | --- | --- | --- | --- | --- | --- | --- | --- | --- | --- | --- | --- | --- | --- | --- | --- | --- | --- | --- | --- | --- | --- | --- | --- | --- |
|  | **n_M13_=105 641**  **n_M2_=76 101** | | **n_M13_=19 149**  **n_M2_=13 951** | | | **n_M13_=27 164**  **n_M2_=19 759** | | | **n_M13_=3 320**  **n_M2_=2 375** | | | **n_M13_=5 092**  **n_M2_=3 349** | | | **n_M13_=7 495**  **n_M2_=5 451** | | **n_M13_=19 706**  **n_M2_=13 834** | | **n_M13_=21 857**  **n_M2_=15 015** | | | **n_M13_=56 340**  **n_M2_=37 992** | | **n_M13_=37 491**  **n_M2_=24 247** | | | **n_M13_=13 050**  **n_M2_=9 497** | | | **n_M13_=16 722**  **n_M2_=11 782** | | |  |
|  | HR | 99% CI | HR | 99% CI | HR | | 99% CI | HR | | 99% CI | HR | | 99% CI | HR | | 99% CI | HR | 99% CI | | HR | 99% CI | HR | 99% CI | | HR | 99% CI | | HR | 95% CI | | HR | 99% CI | |
| **Model 1** |  |  |  |  |  | |  |  | |  |  | |  |  | |  |  |  | |  |  |  |  | |  |  | |  |  | |  |  | |
| **0-6 months** |  |  |  |  |  | |  |  | |  |  | |  |  | |  |  |  | |  |  |  |  | |  |  | |  |  | |  |  | |
| Crude | 1.20 | 1.17-1.22 | 1.22 | 1.16-1.29 | 1.18 | | 1.13-1.23 | 1.00 | | 0.88-1.13 | 1.61 | | 1.48-1.76 | 1.06 | | 0.97-1.15 | 1.31 | 1.25-1.38 | | 1.29 | 1.23-1.35 | 1.32 | 1.28-1.36 | | 1.35 | 1.31-1.40 | | 1.14 | 1.07-1.21 | | 1.40 | 1.33-1.47 | |
| Adjusted | 1.15 | 1.12-1.17 | 1.17 | 1.12-1.23 | 1.15 | | 1.11-1.20 | 1.11 | | 0.97-1.26 | 1.56 | | 1.43-1.71 | 0.99 | | 0.91-1.08 | 1.16 | 1.11-1.22 | | 1.23 | 1.19-1.30 | 1.25 | 1.22-1.29 | | 1.31 | 1.26-1.35 | | 1.16 | 1.09-1.23 | | 1.22 | 1.16-1.29 | |
| **6-11 months** |  |  |  |  |  | |  |  | |  |  | |  |  | |  |  |  | |  |  |  |  | |  |  | |  |  | |  |  | |
| Crude | 1.40 | 1.12-1.16 | 1.14 | 1.10-1.19 | 1.12 | | 1.09-1.16 | 0.98 | | 0.89-1.07 | 1.61 | | 1.50-1.73 | 0.96 | | 0.90-1.02 | 1.26 | 1.21-1.30 | | 1.23 | 1.19-1.28 | 1.22 | 1.20-1.25 | | 1.26 | 1.22-1.29 | | 1.06 | 1.01-1.10 | | 1.32 | 1.27-1.37 | |
| Adjusted | 1.10 | 1.08-1.11 | 1.10 | 1.06-1.14 | 1.10 | | 1.06-1.13 | 1.06 | | 0.97-1.16 | 1.56 | | 1.45-1.68 | 0.90 | | 0.84-0.95 | 1.12 | 1.08-1.16 | | 1.19 | 1.15-1.23 | 1.17 | 1.14-1.19 | | 1.22 | 1.18-1.25 | | 1.06 | 1.02-1.11 | | 1.16 | 1.12-1.21 | |
| **1-2 years** |  |  |  |  |  | |  |  | |  |  | |  |  | |  |  |  | |  |  |  |  | |  |  | |  |  | |  |  | |
| Crude | 1.20 | 1.17-1.22 | 1.21 | 1.16-1.27 | 1.18 | | 1.14-1.23 | 1.16 | | 1.04-1.30 | 1.74 | | 1.57-1.93 | 1.02 | | 0.95-1.10 | 1.39 | 1.32-1.46 | | 1.43 | 1.36-1.49 | 1.46 | 1.42-1.50 | | 1.57 | 1.52-1.63 | | 1.17 | 1.11-1.24 | | 1.46 | 1.38-1.54 | |
| Adjusted | 1.16 | 1.13-1.18 | 1.18 | 1.12-1.23 | 1.16 | | 1.11-1.21 | 1.22 | | 1.09-1.37 | 1.65 | | 1.50-1.83 | 0.96 | | 0.89-1.03 | 1.26 | 1.20-1.33 | | 1.37 | 1.31-1.44 | 1.40 | 1.36-1.44 | | 1.53 | 1.47-1.58 | | 1.17 | 1.11-1.24 | | 1.31 | 1.24-1.38 | |
| **Model 2** |  |  |  |  |  | |  |  | |  |  | |  |  | |  |  |  | |  |  |  |  | |  |  | |  |  | |  |  | |
| **0-6 months** |  |  |  |  |  | |  |  | |  |  | |  |  | |  |  |  | |  |  |  |  | |  |  | |  |  | |  |  | |
| Crude | 1.12 | 1.09-1.15 | 1.15 | 1.08-1.23 | 1.11 | | 1.05-1.17 | 0.87 | | 0.74-1.03 | 1.57 | | 1.40-1.76 | 0.92 | | 0.82-1.03 | 1.21 | 1.14-1.29 | | 1.17 | 1.10-1.25 | 1.24 | 1.20-1.29 | | 1.28 | 1.22-1.34 | | 1.08 | 1.00-1.17 | | 1.30 | 1.21-1.39 | |
| Adjusted | 1.08 | 1.05-1.11 | 1.12 | 1.05-1.19 | 1.10 | | 1.04-1.16 | 0.99 | | 0.84-1.18 | 1.55 | | 1.37-1.74 | 0.87 | | 0.78-0.97 | 1.09 | 1.02-1.16 | | 1.14 | 1.07-1.21 | 1.19 | 1.15-1.24 | | 1.24 | 1.19-1.30 | | 1.11 | 1.02-1.20 | | 1.15 | 1.08-1.23 | |
| **6-11 months** |  |  |  |  |  | |  |  | |  |  | |  |  | |  |  |  | |  |  |  |  | |  |  | |  |  | |  |  | |
| Crude | 1.08 | 1.06-1.10 | 1.09 | 1.04-1.14 | 1.08 | | 1.04-1.12 | 0.90 | | 0.81-1.01 | 1.49 | | 1.36-1.63 | 0.89 | | 0.83-0.96 | 1.18 | 1.13-1.24 | | 1.15 | 1.10-1.20 | 1.16 | 1.13-1.19 | | 1.20 | 1.16-1.24 | | 1.02 | 0.97-1.08 | | 1.22 | 1.17-1.28 | |
| Adjusted | 1.05 | 1.03-1.07 | 1.06 | 1.01-1.11 | 1.07 | | 1.03-1.11 | 1.00 | | 0.89-1.11 | 1.46 | | 1.34-1.60 | 0.84 | | 0.78-0.90 | 1.07 | 1.03-1.12 | | 1.11 | 1.07-1.16 | 1.11 | 1.08-1.14 | | 1.16 | 1.12-1.20 | | 1.04 | 0.98-1.10 | | 1.10 | 1.04-1.15 | |
| **1-2 years** |  |  |  |  |  | |  |  | |  |  | |  |  | |  |  |  | |  |  |  |  | |  |  | |  |  | |  |  | |
| Crude | 1.13 | 1.10-1.15 | 1.17 | 1.11-1.24 | 1.14 | | 1.09-1.19 | 1.09 | | 0.96-1.23 | 1.60 | | 1.42-1.80 | 0.97 | | 0.89-1.05 | 1.31 | 1.24-1.38 | | 1.33 | 1.26-1.40 | 1.37 | 1.32-1.41 | | 1.46 | 1.40-1.53 | | 1.15 | 1.08-1.22 | | 1.36 | 1.28-1.45 | |
| Adjusted | 1.10 | 1.08-1.13 | 1.15 | 1.09-1.21 | 1.13 | | 1.08-1.18 | 1.15 | | 1.01-1.30 | 1.54 | | 1.37-1.72 | 0.91 | | 0.84-0.99 | 1.21 | 1.14-1.27 | | 1.29 | 1.22-1.36 | 1.32 | 1.27-1.36 | | 1.43 | 1.37-1.49 | | 1.15 | 1.08-1.23 | | 1.25 | 1.17-1.32 | |
| **Model 3** |  |  |  |  |  | |  |  | |  |  | |  |  | |  |  |  | |  |  |  |  | |  |  | |  |  | |  |  | |
| **0-6 months** |  |  |  |  |  | |  |  | |  |  | |  |  | |  |  |  | |  |  |  |  | |  |  | |  |  | |  |  | |
| Crude | 1.29 | 1.27-1.32 | 1.27 | 1.21-1.33 | 1.25 | | 1.20-1.30 | 1.09 | | 0.97-1.23 | 1.78 | | 1.64-1.94 | 1.16 | | 1.07-1.25 | 1.46 | 1.40-1.53 | | 1.44 | 1.38-1.50 | 1.40 | 1.37-1.44 | | 1.44 | 1.39-1.48 | | 1.16 | 1.10-1.23 | | 1.54 | 1.47-1.62 | |
| Adjusted | 1.22 | 1.19-1.24 | 1.20 | 1.14-1.26 | 1.21 | | 1.16-1.25 | 1.21 | | 1.07-1.36 | 1.71 | | 1.57-1.86 | 1.07 | | 0.99-1.15 | 1.26 | 1.21-1.32 | | 1.36 | 1.31-1.42 | 1.32 | 1.29-1.36 | | 1.38 | 1.33-1.42 | | 1.17 | 1.10-1.24 | | 1.32 | 1.26-1.39 | |
| **6-11 months** |  |  |  |  |  | |  |  | |  |  | |  |  | |  |  |  | |  |  |  |  | |  |  | |  |  | |  |  | |
| Crude | 1.16 | 1.14-1.18 | 1.15 | 1.11-1.20 | 1.13 | | 1.10-1.17 | 0.99 | | 0.91-1.09 | 1.70 | | 1.58-1.83 | 0.98 | | 0.92-1.04 | 1.28 | 1.24-1.33 | | 1.27 | 1.22-1.31 | 1.25 | 1.22-1.28 | | 1.28 | 1.25-1.32 | | 1.07 | 1.02-1.12 | | 1.34 | 1.29-1.39 | |
| Adjusted | 1.11 | 1.09-1.13 | 1.11 | 1.07-1.15 | 1.11 | | 1.07-1.14 | 1.08 | | 0.98-1.18 | 1.64 | | 1.53-1.77 | 0.91 | | 0.86-0.97 | 1.14 | 1.10-1.18 | | 1.22 | 1.18-1.26 | 1.19 | 1.16-1.21 | | 1.24 | 1.21-1.27 | | 1.07 | 1.03-1.12 | | 1.18 | 1.13-1.22 | |
| **1-2 years** |  |  |  |  |  | |  |  | |  |  | |  |  | |  |  |  | |  |  |  |  | |  |  | |  |  | |  |  | |
| Crude | 1.21 | 1.18-1.23 | 1.23 | 1.17-1.29 | 1.19 | | 1.14-1.24 | 1.16 | | 1.04-1.30 | 1.78 | | 1.61-1.97 | 1.03 | | 0.96-1.10 | 1.41 | 1.34-1.48 | | 1.43 | 1.37-1.50 | 1.49 | 1.45-1.54 | | 1.63 | 1.57-1.69 | | 1.18 | 1.11-1.25 | | 1.47 | 1.39-1.55 | |
| Adjusted | 1.17 | 1.14-1.19 | 1.19 | 1.13-1.25 | 1.17 | | 1.12-1.22 | 1.23 | | 1.09-1.37 | 1.69 | | 1.53-1.87 | 0.96 | | 0.89-1.03 | 1.28 | 1.22-1.35 | | 1.38 | 1.32-1.45 | 1.43 | 1.39-1.48 | | 1.58 | 1.52-1.64 | | 1.18 | 1.12-1.25 | | 1.32 | 1.25-1.39 | |

**Model 1** Original model.

**Model 2** Sensitivity analysis where offspring with an in-patient diagnosis for infection before 2 years of age (A00-B99, G00-G03, J00-J22, K35, L08, N10, N30) were excluded.

**Model 3** Sensitivity analysis where offspring with an in-patient diagnosis for infection before 2 years of age (A00-B99, G00-G03, J00-J22, K35, L08, N10, N30) were categorized as exposed to an antibiotic drug during the infection diagnosis. The time of the first in-patient infection diagnosis was considered.

Any F diagnosis means any ICD-10 F diagnosis not restricted to the following diagnoses:

ICD-10 F30-39, F92 (1.7%) Mood disorders; F40-43, F93 (2.4%) Anxiety disorders; F50 (0.29%) Eating disorders; F51 (0.42%) Nonorganic sleep disorders; F84 (0.68%) Autism Spectrum Disorder (ASD); F90-91 (1.7%) Attention Deficit Hyperactivity Disorder (ADHD) and Conduct disorders; F98 (1.9%) Other behavioral and emotional disorders with onset usually occurring in childhood and adolescence.

Psychotropic medications studied defined according to the Anatomic Therapeutic Chemical (ATC) classification system were found for 4.7% of the cohort and included the following: ATC groups N05 (3.0%) antipsychotics, anxiolytics, hypnotics and sedatives; ATC group N06A (1.2%) antidepressants; ATC group N06B (1.5%) stimulants.

Crude: model without covariates. Adjusted: model adjusted for: maternal age, parity, maternal smoking during pregnancy [yes/no], mother unmarried [yes/no], mother born elsewhere than Finland [yes/no], caesarean section [yes/no], mother’s inpatient care due to mental health disorders [yes/no], mother’s purchase of psychotropic drugs (N05 or N06) during pregnancy [yes/no], mother’s diagnoses related to systemic inflammatory disorders [yes/no], multiple birth [yes/no], offspring sex, perinatal health problems (birth weight < 2500 grams, gestational age < 37 weeks or small for gestational age according to Finnish sex-specific standards) [yes/no], and mother’s prescriptions for antibiotics during pregnancy [yes/no].

The risk estimated in Model 1-3 of Table S4 differ slightly from those in Table 3 because outcomes are in Table S4 recorded from birth until 2014, including also the first 2 years of life. Only diagnoses of sleep disorders (F51) and other behavioral and emotional disorders (F98) occurred before 2 years of age (61% and 20% respectively).
